# Development and Preliminary Validation of the Child & Adolescent Social Cognitions Questionnaire

**DOI:** 10.1007/s10578-021-01163-0

**Published:** 2021-04-03

**Authors:** Eleanor Leigh, David M. Clarkh

**Affiliations:** 1 Department of Experimental Psychology, University of Oxford, Oxford, UK; 2 OxCADAT, The Old Rectory, Paradise Square, Oxford OX1 1TW, UK

**Keywords:** Social anxiety, Youth, Social anxiety-related cognition, Scale

## Abstract

Negative cognitions play a central role in adolescent social anxiety, and yet there is a lack of empirically validated measures assessing these in detail. This study describes the adaptation of the Child & Adolescent Social Cognitions Questionnaire (CASCQ) from the adult version of the scale and its preliminary validation in a general adolescent school sample (N = 671). Exploratory and confirmatory factor analysis on split halves of the data indicated two factors, labelled ‘negative self-concept’ and ‘anxious appearance’, provided the best fit. Totals and subscales possessed good internal consistency and convergent validity. Findings suggest that the CASCQ is a reliable and valid measure of social anxiety-related cognitions in youth and may be useful for research and clinical purposes. Further examination of the scale with pre-adolescents and clinical samples is warranted.

## Introduction

The question of why some individuals continue to feel anxiety in social and performance situations, even in the absence of negative feedback, is critical for understanding the maintenance of social anxiety disorder (SAD) and for efforts to effectively intervene. Cognitive behavioural models suggest that negative thoughts about oneself and others’ reactions in social situations, such as “*people will see that I am nervous*”, “*I will blush*”, and “*I am boring*”, play a central role in the disorder [1–4].

In adults, various scales for assessing such thoughts have been developed, including the Social Cognitions Questionnaire (SCQ) [5]. The SCQ was developed in order to quantify social anxiety-related cognitions [6]. It is comprised of 22 items, each reflecting a common social anxiety-related cognition (e.g. “*People will stare at me*”). Items are rated on two dimensions: how frequently the thought occurs in social situations, and how strongly the thought is believed. In adults, the scale has been shown to have good internal consistency [7] and test–retest reliability [8]. A three or four factor structure was indicated in preliminary work of Clark [7], with items loading onto the first factor clustering around the concept of negative self-concept (e.g. “*I am unlikeable*”), and the second and third factors reflecting concerns about the appearance of anxiety (e.g.“*I will be unable to speak*” in factor 2 and “*I am going red*” in factor 3). The fourth factor, reflecting inferiority, accounted for a relatively small amount of variance and had only two strong item loadings. Finally, treatment studies have shown large reductions in frequency and belief ratings on the SCQ over treatment [9, 10], indicating that the scale is sensitive to change.

SAD usually begins in childhood or adolescence, with a median age of onset of 13 years [11], and therefore there is theoretical and practical importance to understanding its persistence in youth. Recent research indicates that in young people as in adults, negative social anxiety-related cognitions are associated with social anxiety and appear to be relevant to development and maintenance [12–14]. Furthermore, two treatment studies of Cognitive Therapy for adolescent SAD, a treatment designed specifically to target negative social anxiety-related cognitions and beliefs, found significant reductions in the strength of these by the end of treatment [15, 16], consistent with cognitive behavioural accounts of the disorder.

Surprisingly, only two measures of social-evaluative cognition have been evaluated for use with young people. The Child Automatic Thoughts Questionnaire [17, 18] was designed to measure a range of negative thoughts across anxiety domains, and includes 10 items relating to social threat, for example “*other kids are making fun of me*”. The scale has been shown to be reliable and valid, however as pointed out by Wong and colleagues [19], the scale measures ‘in the moment’ negative automatic thoughts but it does not assess broader social-evaluative beliefs. The Report of Youth Social Cognitions is a 14-item measure that was developed recently to examine social-evaluative beliefs in youth [19], including items such as “*other kids think I’m silly*”. Whilst the scale was shown to have good psychometric properties [19], it does not include items capturing the particular ways socially anxious youth fear they will behave in social situations (e.g. “*I will go red*”, “*I will stumble over my words*”). These cognitions are a central feature of cognitive behavioural models of social anxiety [14] and a target for therapies based on these models. In adults, the SCQ measures these cognitions, as well as broader social-evaluative beliefs such as “*I am boring*”, and we think it would be a useful tool to adapt for youth. In particular, it could improve our theoretical understanding of youth social anxiety and aid the delivery of psychological prevention and treatment approaches for SAD in young people.

With this study, we aimed to adapt the SCQ for young people and provide a preliminary examination of its psychometric properties in a school-based sample of UK adolescents. The first aim of the study was to examine the factor structure of the scale. The second aim was to determine preliminary psychometric properties of the scale, in particular internal consistency and construct validity. Construct validity was assessed by examining the associations between the adapted SCQ and scores on a measure of social anxiety, with and without controlling for depression and for generalised anxiety disorder (GAD) symptoms, both of which are strongly associated with social anxiety symptoms [20]. The third aim was to explore and compare scores on the adapted SCQ for older and younger adolescents and for males and females. This is because social anxiety symptoms are known to increase with age during adolescence [21] and to be higher amongst females than males [22] and so we were interested to observe whether scores on the adapted SCQ showed similar gender- and age-related differences.

## Method

### Participants

In total, 671 adolescents aged 11–18 years completed all items of the adapted SCQ as part of their participation in school-based studies of social anxiety in youth. Sample 1 comprised 482 young people in UK school years 7–9 recruited from two non-selective and state-funded secondary schools (aged 11–14 years, mean age = 12.98, SD = 0.87, 248 [51.45%] female). Sample 2 comprised 189 young people in years 12 and 13 attending a sixth form college in the UK (aged 16–18 years, mean age = 17.11, SD = 0.72, 109 [57.67%] female).

### Procedures

The study procedures were in accordance with ethical standards and were approved by the Central University Research Ethics Committee at the University of Oxford (Reference number: R54283/RE001 for Sample 1) and the Psychiatry, Nursing and Midwifery Research Ethics Subcommittee at King’s College London (HR-18/19-8278; Sample 2). For participants under 16 years of age, written informed assent was obtained from all participants and opt-out consent was provided by participants’ parents/guardians. For participants over the age of 16 years, written informed consent was obtained from young people.

Before data collection, the research team explained the study to students during tutor time and information packs were distributed to caregivers and students. All students able to read and write English were encouraged to participate. Data collection took place during school time at least 2 weeks after information about the project had been disseminated. Participants completed a self-report questionnaire pack, including the Social Cognitions Questionnaire adapted for children and adolescents (details below) and measures evaluating social anxiety, anxiety and depression symptoms. For Sample 1, all students in years 7–9 in the two participating schools were invited to participate. 718 students were invited to participate, and the consent rate was 87.2%. For Sample 2, information about the number of students invited was not collected. Of the total 865 participants who participated (n = 657 from Sample 1 and n = 208 from Sample 2), 671 had completed all items of the CASCQ. Those who had completed all items of the CASCQ were more likely to have been from Sample 2 (and therefore older) than Sample 1, compared to those who did not complete all items, but there no differences in gender, social anxiety symptoms, or depression symptoms.

### Measures

*Child & Adolescent Social Cognitions Questionnaire* (CASCQ) is a self-report questionnaire, with each item corresponding to a common social anxiety-related cognition. All 22 items from the adult Social Cognitions Questionnaire [5] were included in the youth version, with wording adapted to be suitable for children and adolescents. In consultation with experts in the field (CC and PW), six additional items were added which were considered to be relevant to young people, for example, “*People will be angry with me*”. A sample of five young people who had recovered from SAD after treatment reviewed the items (original and revised, and the additional items) and rated each one from 0 to 5 on how understandable they were. Revisions improved ratings of all items. The pool of 28 items were all rated 4/5 or 5/5. Respondents are asked to rate how frequently they experience the thought when they are socially anxious, from 1 (never) to 5 (every time), and how much they believe the thought when it occurs, from 0 (not at all) to 100 (totally). Two scores are generated: a mean frequency score and mean belief score. The final 27 item scale is freely available at https://oxcadatresources.com.

*The Liebowitz Social Anxiety Scale for Children and Adolescents-Self Report version* (LSAS-CA-SR; [23]) is a 24-item self-report scale measuring social anxiety in young people aged 7–18 years old. This scale assesses levels of both fear and avoidance in social and performance situations. Each item has a 0–3 rating represented by ‘none’, ‘mild’, ‘moderate’ and ‘severe’. The total score ranges from 0 to 144. The scale has acceptable reliability and validity [23]. The internal consistency in the current sample was α = 0.97.

*The Short Mood and Feelings Questionnaire* (SMFQ; [24]) is a 13-item self-report questionnaire designed to assess different aspects of depressive symptoms in young people aged 6–17 years. Each item ranges from 0 (not true) to 2 (true). Total scores were obtained by summing up all the items. This scale has acceptable reliability and validity [25]. The internal consistency in this sample was α = 0.91.

The child version of the *Screen for Anxiety and Related Disorders* (SCARED; [26]) is a 41-item self-report questionnaire used to screen for childhood anxiety disorders including general anxiety disorder, separation anxiety disorder, panic disorder, social anxiety disorder, and school phobia. The GAD subscale was used to examine validity in the present study. The scale has good reliability and validity [26, 27]. Internal consistency of the total scale in this sample was α = 0.96.

### Data Analysis

Data was prepared in SPSS v. 26 and analysis was conducted in R [28] using the lavaan package [29]. Mean substitution was performed when less than 10% of items were missing in the LSAS-CA-SR, SCARED and SMFQ questionnaires [30]. At variable level, missing data was low, ranging from 1.19 to 4.32% (LSAS-CA-SR was missing for n = 8 participants, SCARED for n = 29, and SMFQ for n = 20). Little’s MCAR test indicating that data was missing completely at random at variable level (*p* > 0.05). For analysis that involved the LSAS-CA-SR, SMFQ and SCARED (i.e. examination of validity), pairwise deletion was used for data missing at variable level.

The factor structure of the CASCQ was examined using a combination of Exploratory Factor Analysis (EFA) and Confirmatory Factor Analysis (CFA) in the general adolescent sample. The sample was split in two randomly (0.6/0.4 ratio). EFA was conducted in the first half of the dataset and then this factor structure was confirmed in the second half using CFA.

The factor structure was examined separately for belief and frequency ratings. The frequency scale generates ordinal data, and so the Ordinary Least Squared method of extraction was used. For the belief scale, the Kolmogorov–Smirnov test showed that the data was normally distributed and so the maximum likelihood method of extraction was used. For both EFA an oblique rotation was used (Oblimin), because we expected the factors to be correlated with each other. To determine the appropriate number of factors to retain we used parallel analysis (for non-normal data in the case of frequency ratings, and normal data in the case of belief ratings) and we examined the scree plot and interpretability of the generated factors. In addition, we examined item loadings, and in line with Guadagnoli and Velicer [31], considered factors on which four or more items had loadings of at least 0.6 as reliable. In terms of evaluating item loadings onto factors, we were guided by Stevens [32], who suggested using a cut-off of 0.4. To test the goodness of fit of the models in the CFA, we examined the Standardized Root Mean Square Residual (SRMR), the Root Mean Square Error of Approximation (RMSEA), the Comparative Fit Index (CFI) and the Tucker Lewis Index (TLI). SRMR values below 0.08 and RMSEA values less than 0.06 indicate a good fit. CFI and TLI values of 0.90 or greater suggest an acceptable fit [33, 34].

The rest of the analysis was undertaken with the whole sample. First, mean scores were calculated for the total score and for the subscales derived from the indicated factor structure. Then, internal consistency of the total score and subscales was assessed with Cronbach’s alpha, with alpha greater than 0.70 indicating acceptable consistency and above 0.80 good consistency [35]. To examine construct validity, correlations between the CASCQ totals and subtotals and the LSAS-CA-SR were examined, before carrying out partial correlations between the CASCQ totals and subtotals and the LSAS-CA-SR, controlling for GAD symptoms (from the SCARED) and then for depression symptoms (SMFQ). Finally, we examined age and gender differences in CASCQ-F and CASCQ-B total and subtotal mean scores.

## Results

### Exploratory Factor Analysis: Belief Ratings

**Fig. 1:**
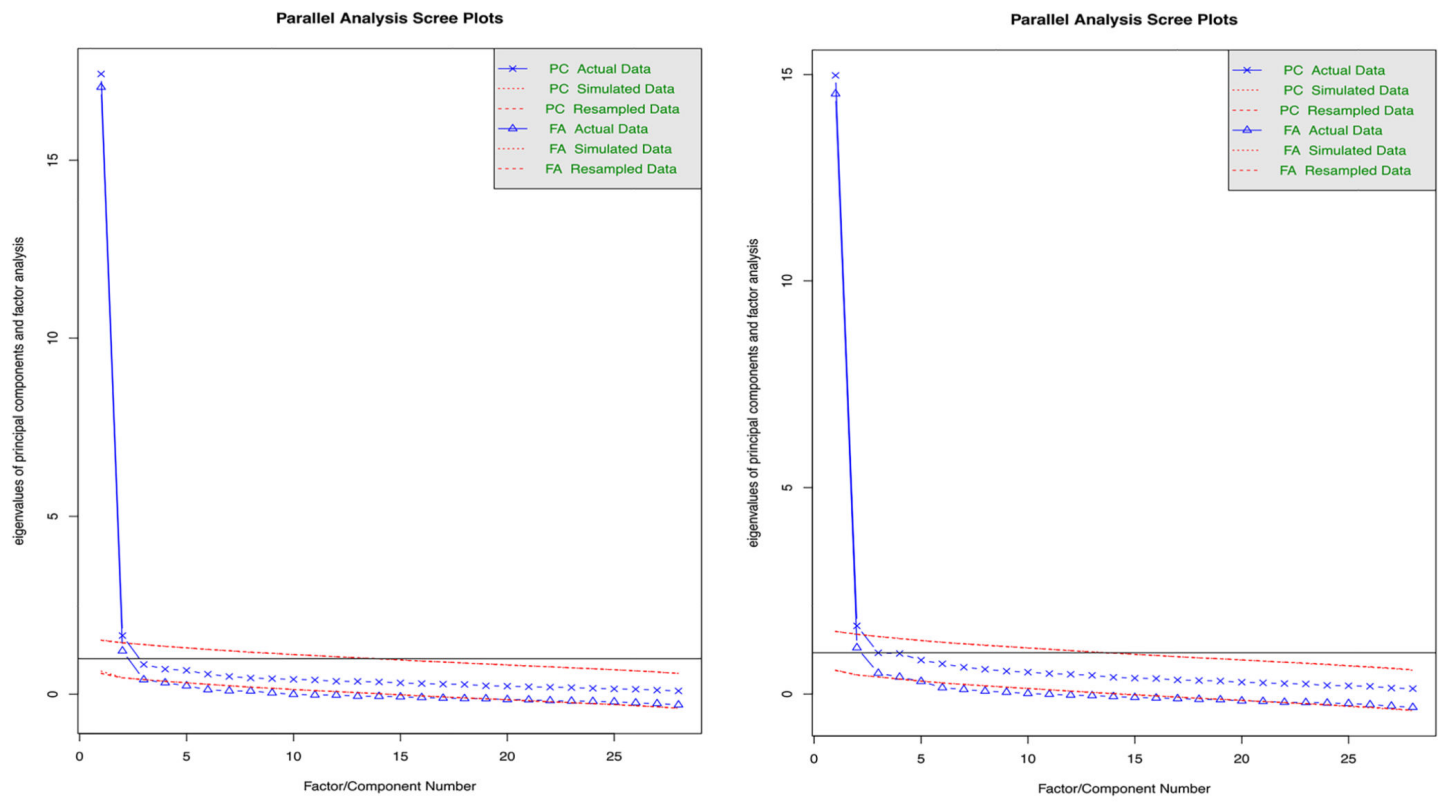
Scree plots of indicated factors to extract for CASCQ Belief data (left hand plot) and CASCQ Frequency data (right hand plot)

The Kaiser–Meyer–Olkin measure of sampling adequacy (KMO = 0.98) and Bartlett’s test of sphericity (χ^2^(378) = 17853.36, p < 0.001) indicated that the data was suitable for factor analysis. Parallel analysis and examination of the scree plot (Fig. 1; left hand plot) suggested that two factors were appropriate for extraction.

The two-factor solution explained 65% of the variance, with the first factor explaining 39% and the second explaining 26%. Standardized factor loadings are shown in Table 1. Fifteen items loaded onto Factor 1, 13 of these had a loading of ≥ 0.60. Thirteen items loaded onto Factor 2. Seven of these had a loading of ≥ 0.60. The two factors were correlated (*r* = 0.76), which supports the idea that the two factors reflect related but not identical concepts. Factor 1 reflects ‘negative self-concept’ whilst Factor 2 represents ‘anxious appearance’.

### Exploratory Factor Analysis: Frequency Ratings

The Kaiser–Meyer–Olkin measure of sampling adequacy (KMO = 0.97) and Bartlett’s test of sphericity (χ^2^(378) = 13,989.21, p < 0.001) indicated that the data was suitable for factor analysis. Parallel analysis suggested that three factors were appropriate for extraction, examination of the scree plot (Fig. 1; right hand plot) indicated that between two and three factors were appropriate, and on examination of the factor loadings and interpretability, a two factor solution was decided upon.

The two factor solution explained 56% of the variance, with the first factor explaining 35% and the second explaining 21%. Standardized factor loadings are shown in Table 2. Sixteen items loaded onto Factor 1, twelve with a loading of ≥ 0.60. Twelve items loaded onto Factor 2. Six of these had a loading of ≥ 0.60. The two factors were correlated (*r* = 0.76), which supports the idea that the two factors reflect related but not identical concepts. Factor 1 reflects ‘negative self-concept’ whilst Factor 2 represents ‘anxious appearance’.

### Confirmatory Factor Analysis

Based on the EFA, item 25 (“*I will get picked on and teased*”) was dropped from the scale before the CFA, due to instability of loading. This is because the item loaded onto Factor 2 for Belief ratings (loading for Factor 1: 0.38; loading for Factor 2: 0.44), but onto Factor 1 for Frequency ratings (loading for Factor 1: 0.53; loading for Factor 2: 0.14). Loadings from the CFA are shown in Table 3; loadings of all items for the Belief model and the Frequency model were 0.60 or above.

For the Belief ratings, SRMR (0.05), CFI (0.90) and TLI (0.90) were acceptable, but RMSEA was above the recommended threshold (RMSEA = 0.09 (95% confidence interval [CI]: 0.086, 0.098)). The fit of the model for the Frequency ratings was better, with all indices in the acceptable range. SRMR was 0.05, RMSEA was 0.05 (95% CI: 0.042–0.057), and CFI and TLI were both above 0.90 (CFI = 0.998 and TLI = 0.998).

### Internal Consistency

As can be seen in Table 4, internal consistency for the total scores and subscales was good for all frequency and belief scales, ranging from 0.90 to 0.98.

### Convergent Validity

Convergent validity of the CASCQ was inspected by examining the association between the total and subtotals (for both frequency and belief scales) with self-report measures of social anxiety (LSAS-CA-SR). As expected, and shown in Table 5, each subscale and the totals for both frequency and belief ratings were significantly and positively correlated with the LSAS-CA-SR total (*r*s > 0.45). To rule out the possibility that the association between the CASCQ and social anxiety symptoms is largely due to comorbid GAD or depression symptoms, the correlations between the CASCQ subtotals and totals and LSAS-CA-SR total were repeated first controlling for depression symptoms (Belief Total: *partial* r = 0.336, *p* < 0.01; Frequency Total: *partial* r = 0.580; *p* < 0.01), and second controlling for GAD symptoms (Belief Total: *partial* r = 0.300, *p* < 0.01; Frequency Total: *partial* r = 0.486; *p* < 0.01). Correlations with Belief and Frequency subscale scores also remained significant when controlling for depression symptoms and for GAD symptoms (*p* < 0.01).

### Age and Gender Analysis

As can be seen in Table 4, in the general adolescent samples, girls scored more highly than boys and the older adolescents scored more highly than the younger adolescents on all totals and subtotals of the CASCQ. The same pattern of results is reflected in the LSAS-CA-SR scores, with higher LSAS-CA-SR scores in girls (*p* < 0.001) and older adolescents (*p* < 0.001).

## Discussion

This study provides a preliminary psychometric analysis of the Social Cognitions Questionnaire [6], adapted for children and adolescents (CASCQ). We found support for the reliability and validity of the scale in a general sample of UK adolescents.

We were initially interested in examining the factor structure of the scale. We undertook EFA in a random half of the sample and identified a two-factor solution for both belief and frequency ratings. Item loadings were strong and broadly consistent across the two scoring forms. The factor structure was then supported in a CFA undertaken in the second half of the sample. The fit was acceptable for the frequency ratings. Whilst certain fit indices were more marginally acceptable for the belief ratings, we note that all item loadings remained high. Inspecting the item loadings, we interpreted one factor as ‘negative self-concept’ and the other as ‘anxious appearance’. Example items loading on the ‘negative self-concept’ factor include “*people will think I am boring*” and “*I am unlikeable*”. Example items loading on the ‘anxious appearance’ factor include “*I will be unable to speak*” and “*I am going red*”. The correlation between the two factors for the Belief rating was *r* = 0.83 and *r* = 0.81 for the Frequency rating. This suggests that the two factors show moderate overlap as we would expect, but they seem to measure slightly different aspects of social anxiety-related cognitions.

Although here we identified two factors compared to the three/four specified in the factor analysis undertaken by Clark [7] in the adult SCQ, there is notable overlap in the apparent underlying constructs. For example, the first factor of the adult SCQ relates to negative self-concept, as we found here, and the second and third factors reflected anxious appearance, as our second factor did. The two factors are consistent with leading cognitive behavioural accounts of social anxiety [1, 3, 4]. These accounts align to suggest that unconditional negative beliefs about the self, such as “*I’m unlikeable*”, lie at the heart of social anxiety, as do cognitions related to social performance deficits and observability of anxious feelings. The finding is important as it supports the suggestion that adult cognitive models of social anxiety, such as that of Clark and Wells [4] are also relevant to youth [12].

Typically measures of beliefs and cognitions in youth anxiety have assessed either the frequency or the strength of the belief. We believe the CASCQ is unusual in measuring both frequency and belief ratings. Each shows substantial correlations with social anxiety. The correlation between frequency and belief (*r* = 0.71) suggests approximately 50% shared variance. Although the two are interrelated, it seems possible that they index slightly different features of cognition and it is therefore worth retaining both. In the present sample the correlation between social anxiety and frequency ratings is somewhat higher than between social anxiety and belief ratings. It would be interesting to examine whether this remains so in a clinical sample.

Encouragingly, the scale appears to be reliable, with excellent internal consistency for all the totals and subtotals. Examination of the patterns of association suggest that as expected, the CASCQ was positively and significantly correlated with a measure of social anxiety, but importantly the correlations with social anxiety symptoms persisted when controlling for depression symptoms and for generalised anxiety symptoms. This indicates that the observed associations are not an artefact of the relationship between symptoms of either depression or generalised anxiety and negative social anxiety-related cognitions.

The finding that socially anxious cognitions were more frequent and strongly held amongst girls compared to boys in our general population sample was as expected given the consistent finding of higher levels of social anxiety in females [36]. Similarly, the finding of an age effect was also expected, with higher levels of socially anxious cognitions in older, compared to younger, adolescents. Epidemiological studies consistently show increasing levels of social anxiety from early adolescence though to early adulthood [36].

The study has a number of limitations. The CASCQ was designed for children and adolescents, but we only recruited adolescents. It will be important to see how the measure performs with pre-adolescents. Our sample was recruited from schools. Future studies with clinic-recruited young people, including those with and without SAD, will be informative in terms of psychometric properties of the scale in a clinical population as well as its sensitivity and specificity. Participants completed the CASCQ at one time point only, and so we do not know how reliable the measure is over time. Finally, we note that our measures were exclusively self-report. We took this decision given the centrality of the internal veracity of these cognitions (rather than objective reality) to the persistence of social anxiety in cognitive behavioural accounts, but the inclusion of parent and/ or teacher report symptom measures would be informative.

The present study describes the development of a measure of social anxiety-related cognitions for children and young people, the CASCQ and evaluation of its psychometric properties. With increasing interest in the adaptation of cognitive behavioural treatments for SAD, such as Cognitive Therapy for youth that have been proven to be highly effective for adult SAD [37], comes the need for questionnaires that measure key treatment targets. Here, we hope to have developed a tool that will be useful to both clinicians and researchers.

## Summary

In this paper, we describe the adaptation of the Child & Adolescent Social Cognitions Questionnaire (CASCQ) from the adult version of the scale and its preliminary validation in a general adolescent school sample. Factor analysis indicated two factors provided best fit, reflecting ‘negative self-concept’ and ‘anxious appearance’. We found evidence for good internal consistency and convergent validity for totals and subtotals. Findings from this preliminary study suggest that the CASCQ is a reliable and valid measure of social anxiety-related cognitions in youth and may be useful for research and clinical purposes.

## Figures and Tables

**Table 1:** Pattern matrix showing the belief item loadings with a two-factor solution

		Factor 1	Factor 2
1	I will be unable to speak	−0.03	**0.79**
2	I am unlikeable	**0.84**	0.01
3	I am going to tremble or shake uncontrollably	−0.04	**0.85**
4	People will stare at me	**0.51**	0.31
5	I am being an idiot	**0.75**	0.08
6	People won’t want to be friends with me	**0.84**	0.04
7	I will be frozen with fear	−0.09	**0.93**
8	I will drop or spill things	0.17	**0.61**
9	I am going to be sick	0.07	**0.69**
10	I am not good enough	**0.92**	−0.08
11	I will babble or talk funny	0.31	**0.49**
12	I am not as good as others	**0.97**	−0.12
13	I will be unable to concentrate	0.32	**0.45**
14	I will be unable to write properly	0.07	**0.73**
15	People are not interested in me	**0.95**	−0.08
16	People won’t like me	**0.96**	−0.06
17	People will make fun of me	**0.79**	0.1
18	I will sweat/perspire	−0.02	**0.74**
19	I am going red	0.10	**0.55**
20	I am weird/different	**0.72**	0.07
21	People will see I am nervous	0.21	**0.54**
22	People think I am boring	**0.63**	0.2
23	I will embarrass myself*	**0.75**	0.11
24	People will be angry with me*	**0.55**	0.3
25	I will get picked on and teased*	0.38	**0.44**
26	I will look stupid*	**0.76**	0.13
27	I will be forced to do things I don’t want to do*	0.22	**0.58**
28	People will laugh at me*	**0.73**	0.12

Item loadings greater than 0.4 are indicated in bold

* Asterisked items are those items added to the original Social Cognitions Questionnaire when adapting the scale for children and young people

**Table 2:** Pattern matrix showing the Frequency item loadings with a two-factor solution

		Factor 1	Factor 2
1	I will be unable to speak	0.11	**0.56**
2	I am unlikeable	**0.92**	−0.09
3	I am going to tremble or shake uncontrollably	−0.08	**0.79**
4	People will stare at me	**0.44**	0.34
5	I am being an idiot	**0.73**	0.07
6	People won’t want to be friends with me	**0.87**	−0.04
7	I will be frozen with fear	−0.01	**0.74**
8	I will drop or spill things	0.11	**0.51**
9	I am going to be sick	0.01	**0.63**
10	I am not good enough	**0.76**	0.08
11	I will babble or talk funny	0.26	**0.47**
12	I am not as good as others	**0.74**	0.08
13	I will be unable to concentrate	0.12	**0.55**
14	I will be unable to write properly	−0.06	**0.67**
15	People are not interested in me	**0.89**	−0.05
16	People won’t like me	**0.99**	−0.16
17	People will make fun of me	**0.76**	0.1
18	I will sweat/perspire	0.04	**0.65**
19	I am going red	0.16	**0.46**
20	I am weird/different	**0.56**	0.22
21	People will see I am nervous	0.17	**0.62**
22	People think I am boring	**0.62**	0.13
23	I will embarrass myself	**0.62**	0.24
24	People will be angry with me	**0.47**	0.27
25	I will get picked on and teased	**0.53**	0.14
26	I will look stupid	**0.69**	0.14
27	I will be forced to do things I don’t want to do	0.19	**0.55**
28	People will laugh at me	**0.65**	0.15

Item loadings greater than 0.4 are indicated in bold

**Table 3:** Item loadings from the CFA for the belief and frequency ratings

	Belief		Frequency
**Negative self-concept factor**			
I am unlikeable	0.86		0.89
People will stare at me	0.75		0.78
I am being an idiot	0.81		0.82
People won’t want to be friends with me	0.87		0.86
I am not good enough	0.89		0.85
I am not as good as others	0.87		0.89
People are not interested in me	0.87		0.91
People won’t like me	0.90		0.94
People will make fun of me	0.88		0.86
I am weird/different	0.73		0.77
People think I am boring	0.83		0.82
I will embarrass myself	0.88		0.86
People will be angry with me	0.79		0.78
I will look stupid	0.85		0.90
People will laugh at me	0.81		0.86
**Anxious appearance factor**			
I will be unable to speak	0.78		0.74
I am going to tremble or shake uncontrollably	0.75		0.75
I will be frozen with fear	0.79		0.74
I will drop or spill things	0.69		0.66
I am going to be sick	0.72		0.65
I will babble or talk funny	0.71		0.79
I will be unable to concentrate	0.72		0.77
I will be unable to write properly	0.71		0.71
I will sweat/perspire	0.77		0.71
I am going red	0.70		0.60
People will see I am nervous	0.81		0.82
I will be forced to do things I don’t want to do	0.79		0.83

**Table 4:** Means and standard deviations and internal consistency of the CASCQ totals and subtotals

	Female (SD) (n = 357)^‡^	Male (SD) (n = 3O5)^‡^	*t*-test (df)	*d*	Age 11–14 years (SD) (n = 482)	Age 16–18 years SD) (n=l89)	*t*-test (df)	*d*	Total sample (SD) (n = 67l)	Cronbach α (95% CI)
*Belief*									
Total	38.66 (26.66)	24.91 (26.35)	*t*(660) = 6.65**	0.52	30.31 (27.10)	37.69 (27.40)	*t*(669) = 3.16**	0.27	32.39 (27.37)	0.98 (0.97, 0.98)
Negative self-concept	44.73 (31.66)	28.13 (29.09)	*t*(660) = 7.14**	0.56	35.10(30.81)	42.41 (30.57)	*t*(669) =2.77**	0.24	37.16 (30.90)	0.97 (0.97, 0.98)
Anxious appearance	31.66 (24.91)	21.19 (25.46)	*t*(660) = 5.34**	0.42	24.79 (25.37)	32.35 (25.85)	*t*(669) = 3.41**	0.29	26.89 (25.71)	0.94 (0.93, 0.94)
*Frequency*									
Total	2.51 (1.02)	1.84 (0.78)	*t*(660) = 9.39**	0.73	2.10 (0.92)	2.47 (1.04)	*t*(669) = 4.48**	0.38	2.21 (0.97)	0.97 (0.96, 0.97)
Negative self-concept	2.80 (1-19)	2.01 (0.95)	*t*(660) = 9.36**	0.73	2.33 (1.12)	2.70 (1.18)	*t*(669) = 3.70**	0.32	2.44(1.15)	0.96 (0.96, 0.97)
Anxious appearance	2.19 (0.93)	1.66 (0.67)	*t*(660) = 8.32**	0.65	1.84 (0.78)	2.21 (0.97)	*t*(669) = 5.21**	0.45	1.94 (0.86)	0.90 (0.89, 0.91)

*SD* standard deviation, *df* degrees of freedom, *CI* confidence interval

* lndicates *p*<0.01;

** Indicates *p*< 0.001

‡ n = 9 participants did not indicate their gender

**Table 5:** Correlations between CASCQ totals and subtotals and social anxiety symptom measure

	CASCQ-B	CASQ-B-N	CASQ-B-A	CASCQ-F	CASQ-F-N	CASQ-F-A
**LSAS-CA-SR**	**0.56** **	**0.59** **	**0.45** **	**0.76** **	**0.74** **	**0.70** **
CASCQ-B		0.97**	0.94**	0.71**	0.68**	0.66**
CASQ-B-N			0.83**	0.74**	0.76**	0.63**
CASQ-B-A				0.57**	0.50**	0.63**
CASCQ-F					0.97**	0.92**
CASQ-F-N						0.81**

*LSAS-CA-SR* Leibowitz Social Anxiety Scale for Children and Adolescents Self-Report Version, *CASCQ-B* Child and Adolescent Social Cognitions Questionnaire Total Belief, *CASCQ-B-N* Child and Adolescent Social Cognitions Questionnaire Negative Self-Concept Belief, *CASCQ-B-A* Child and Adolescent Social Cognitions Questionnaire Anxious Appearance Belief, *CASCQ-F* Child and Adolescent Social Cognitions Questionnaire Total Frequency, *CASCQ-F-N* Child and Adolescent Social Cognitions Questionnaire Negative Self-Concept Frequency, *CASCQ-F-A* Child and Adolescent Social Cognitions Questionnaire Anxious Appearance Frequency

* Indicates *p* < 0.01;

** Indicates *p* < 0.001;

Correlations between the LSAS-CA-SR and CASCQ totals and subscales are shown in bold
